# CFD-driven optimization and experimental validation of venturi-based thrombectomy devices in a circle of willis

**DOI:** 10.1038/s41598-026-52832-w

**Published:** 2026-06-18

**Authors:** Iham F. Zidane, Xiaochen Wang, Hainan He, Xianghong Ma

**Affiliations:** 1 Mechanical Engineering Department, College of Engineering and Technology, Arab Academy for Science, Technology and Maritime Transport, Alexandria, 1029, Egypt; 2 National Engineering Technology Research Center of Flat Rolling Equipment, University of Science and Technology Beijing, Beijing, 100083 China; 3 College of Engineering and Applied Science, Aston University, Aston Triangle, Birmingham, B4 7ET UK

**Keywords:** Circle of willis, Computational fluid dynamics, Mechanical thrombectomy, Aspiration device, Ischemic stroke, Engineering, Medical research

## Abstract

The geometry of the Circle of Willis poses major challenges for mechanical thrombectomy, where device navigability and effective thrombus removal determine treatment success. This study investigated the performance of venturi-inspired aspiration thrombectomy devices in a simplified cerebral artery segment representative of the middle cerebral artery (MCA), a frequent site of occlusion. Five designs (30°, 45°, 60° venturi, 7/11° taper, and cylindrical control) were assessed using a combined computational–experimental framework. On the computational side, unsteady Reynolds-averaged Navier–Stokes (URANS) simulations were performed in ANSYS Fluent 19.2 with k–ε turbulence closure. Blood–clot interactions were modeled using a Volume of Fluid (VOF) multiphase formulation with Carreau–Yasuda non-Newtonian rheology. In vitro, stereolithography-fabricated prototypes were tested with porcine thrombi in silicone arterial phantoms. CFD predicted extraction times of 2.12 s for the control and 1.64 s for the 45° venturi, with efficiency plateauing beyond 45°. Experimental results confirmed this trend, showing the 45° design as optimal and all venturi devices outperforming the control. Fragmentation analysis revealed a trade-off, with the 60° venturi producing more than twice the fragments of the 30°. These findings demonstrate that venturi taper geometry critically influences aspiration efficiency and fragmentation and establish CFD–experiment integration as a foundation for optimizing next-generation thrombectomy devices.

## Introduction

Stroke remains the second leading cause of death worldwide and a primary contributor to long-term disability^1^. According to the Global Burden of Disease (GBD) Study, ischemic stroke accounts for approximately 85% of all stroke cases and continues to increase in prevalence despite advances in preventive and acute care strategies^2^. Over the past decade, endovascular thrombectomy (EVT) has revolutionized the treatment of large-vessel occlusion (LVO), with multiple landmark trials and subsequent meta-analyses demonstrating significantly improved outcomes compared to medical therapy alone^3,4^. Nonetheless, substantial challenges persist. Recanalization failure, thrombus fragmentation, and distal embolization remain common even when using current-generation stent retrievers and aspiration systems^5–7^. These limitations underscore the ongoing need for device innovation, informed by hemodynamic principles and validated through rigorous experimental and computational evaluation.

### The circle of willis: collateral hub and design challenge

The Circle of Willis (CoW) is the brain’s principal arterial collateral system, playing a critical role in redistributing flow during arterial occlusion. However, anatomical studies indicate that a complete CoW, featuring intact anterior and posterior communicating arteries, is present in fewer than 50% of individuals^8,9^. Variations such as hypoplasia or absence of communicating arteries significantly affect compensatory flow pathways and influence ischemic burden and stroke prognosis^10,11^. Functional studies show that the CoW operates as a pressure-dissipating system under physiological conditions but becomes a crucial collateral route during stenosis or occlusion^9,10^. Experimental and computational models have demonstrated that CoW anatomy and vessel caliber strongly modulate embolic trajectories, perfusion heterogeneity, and thrombectomy outcomes^5,8^.

Beyond static anatomical classification, recent CFD studies have demonstrated that even subtle variations in CoW geometry can profoundly alter collateral flow patterns and shear stress distributions under pathological states^12–14^. Patient-specific modeling across hundreds of subjects has enabled functional phenotyping of CoW integrity and its relationship to embolic risk^13–15^. MR angiography and hemodynamic modeling have further shown how variations in communicating artery caliber affect posterior collateral sufficiency and anterior circulation compensation^11,16^.

Several large-scale studies, such as those by Vrselja et al.^9^ and Alastruey et al.^8^, highlighted how anatomical variants of the CoW alter cerebral perfusion and collateralization. More recent studies, including Sriwastwa et al.^5^, have linked CoW variants with stroke outcomes following revascularization therapy. Advanced imaging and simulation methods (Hendrikse et al.^10^; Behland et al.^13^) continue to refine our understanding of CoW physiology and pathophysiology, reinforcing the importance of incorporating CoW variation in both clinical and computational thrombectomy studies**.**

### Aspiration thrombectomy and device design considerations

Direct aspiration thrombectomy has emerged as a practical and efficient alternative to stent retrievers, offering procedural simplicity, lower device cost, and favorable clinical outcomes in select patients. However, aspiration success depends on a complex interplay of factors, including catheter inner diameter, tip geometry, applied vacuum pressure, thrombus composition, and alignment with the thrombus^17,18^. While bench-top and clinical studies have shown that larger-bore aspiration catheters improve first-pass effect and recanalization rates, increased stiffness may compromise deliverability in tortuous vessels^12^.

Physics of thrombus ingestion remains a subject of ongoing investigation. Turk et al.^19^ demonstrated that suction-based thrombus retrieval, particularly with the direct aspiration first-pass technique (ADAPT), can achieve high rates of successful recanalization with reduced procedure times. Jakobek et al.^20^ further showed in vitro that catheter position relative to collateral pathways significantly alters aspiration performance and embolic clearance. Together, these findings reinforce the importance of catheter geometry, positioning, and suction force in optimizing aspiration thrombectomy design and outcomes.

### Computational modeling in thrombectomy research

Computational modeling is a crucial tool in thrombectomy research, enabling in silico analysis of mechanical procedures. This approach allows researchers to simulate the interaction between devices and blood thrombuss, considering factors like thrombus composition and material properties. For example, Fregona et al.^21^ showed that red blood cell-rich thrombuss are more prone to fragmentation. Bridio et al.^22^ developed a low-dimensional surrogate model for rapid strain estimation, while Luraghi et al.^23^ evaluated the performance of combined stent-retriever and aspiration techniques. Other work has focused on creating patient-specific models of the cerebral vasculature^24^, proposing new models for thrombus deformation during aspiration^25^, and modeling the effects of microthrombi on tissue oxygenation^26^. This research, which also includes a consensus-based review on using these methods for regulatory purposes^27^, demonstrates that computational modeling is essential for improving our understanding of thrombectomy mechanics and developing better devices and strategies.

### Beyond laminar flow: turbulence and blood rheology

While blood flow in cerebral arteries is often modeled as laminar, recent studies challenge this assumption in the context of complex geometries and device-induced disturbances. Saqr and Zidane^28^ identified non-Kolmogorov turbulence regimes in arterial flow, characterized by inverse energy cascades with potential implications for endothelial mechanobiology. Such turbulence may emerge in aneurysmal sacs, near bifurcations, or during active aspiration.

Additionally, the non-Newtonian nature of blood must be accounted for in CFD simulations. The Carreau–Yasuda model has been validated as a physiologically realistic representation of shear-dependent blood viscosity, outperforming Newtonian approximations^29,30^.

The importance of considering both turbulence and non-Newtonian effects in blood flow simulations, initially highlighted by Saqr and Zidane^28^, has been further underscored by recent research. Studies such as those by Cheng et al.^31^ and Calzetta et al.^32^ have advanced our understanding of turbulence and its energy spectrum in physiological and non-Newtonian fluids. Furthermore, Garven et al.^33^ and Chauhan et al.^34^ have demonstrated these principles in specific biomedical applications, including multiscale modeling for pediatric cardiovascular systems and the hemodynamics of bileaflet mechanical heart valves, respectively. These collective findings validate the ongoing efforts to develop more physiologically realistic models.

### Novelty and study objectives

A critical gap in current thrombectomy research lies in the lack of integrated approaches that combine CFD optimization with experimental validation in physiologically realistic environments. The present study aims to address this gap by evaluating venturi-inspired aspiration thrombectomy device geometries using both CFD and in-vitro testing in a Circle of Willis model.

In addition to clot extraction efficiency, the present study also evaluates visible clot fragmentation in the in-vitro experiments as a secondary outcome, since fragmentation may influence the practical safety and effectiveness of aspiration thrombectomy.

The CFD framework employed Unsteady Reynolds-Averaged Navier–Stokes (URANS) simulations in ANSYS Fluent 19.2 to solve the governing equations of incompressible, unsteady flow in a simplified cerebral artery segment representative of the CoW, focusing on the middle cerebral artery (MCA), a frequent site of occlusion. A Volume of Fluid (VOF) multiphase model was implemented to capture blood–thrombus interactions, with blood modeled as a non-Newtonian fluid via the Carreau–Yasuda law and thrombi represented as highly viscous deformable occluding phases positioned downstream of the catheter tip within the VOF framework. Turbulence was modeled using the standard k–ε closure within a URANS framework. While this approach does not resolve turbulence spectra, flow instabilities observed in the simulations are later interpreted in light of recent reports of transitional turbulent kinetic energy cascades in cerebral arteries^28^, providing a physiologically relevant context for understanding aspiration-induced unsteadiness.

The objectives were:

- To evaluate multiple venturi geometries (30°, 45°, 60° curved; 7/11° straight; plus a control design) and quantify their influence on flow dynamics and thrombus extraction efficiency in the CFD model, while assessing fragmentation experimentally in the in-vitro tests.
- To validate Computational Fluid Dynamics (CFD) predictions, which utilize an Eulerian-Eulerian approach, against experimental results using porcine thrombus models; and
- To assess the performance of various designs of aspiration-based thrombectomy devices within a Circle of Willis phantom. The study emphasizes their extraction efficiency in the middle cerebral artery (MCA), which is a key component of the CoW that branches off the internal carotid artery and is a frequent location for thrombus occlusion.

## Methods

Five designs of aspiration based thrombectomy device (30°, 45°, 60° curved, 7/11° straight, and a cylindrical control) were investigated through computational fluid dynamics (CFD) simulations and bench-top experiments. Simulations were performed in ANSYS Fluent 19.2 using the URANS framework with the standard k–ε turbulence closure. The choice of a URANS turbulence closure was motivated by the pulsatile, aspiration-driven, and geometry-disturbed nature of the present flow, together with previous reports indicating that cerebral hemodynamics may depart from purely laminar behavior and may exhibit transitional, non-classical turbulent features under complex unsteady conditions. Within this framework, the standard k–ε model was selected as a robust and computationally efficient two-equation closure for the present comparative parametric study, in which all device geometries were evaluated under identical operating conditions. The primary objective of the CFD analysis was to establish the comparative ranking of device performance, rather than to resolve detailed near-wall turbulence structure or turbulence spectra. In this context, the standard k–ε model was considered appropriate because of its established robustness, stable convergence behavior, and wide use in mean-flow engineering simulations. While the k–ω SST model is an important alternative for flows with stronger adverse pressure gradients, separation, and enhanced near-wall sensitivity, a single turbulence-model framework was retained across all simulated geometries to ensure direct comparison of device-performance trends^35,36^. This model captures mean unsteady separation and shear but does not resolve turbulence spectra; spectral interpretations are deferred to the Discussion. A Volume of Fluid (VOF) multiphase model was implemented to capture blood–thrombus interactions: blood was treated as a Carreau–Yasuda non-Newtonian fluid, while thrombi were represented as highly viscous deformable occluding phases positioned downstream of the catheter tip. The Courant number was maintained below unity, and a time step of 0.001 s was used to resolve the pulsatile inlet waveform and turbulence-like fluctuations. In parallel, experimental validation was performed using porcine blood thrombus in silicone tube. This model represented a simplified segment of the middle cerebral artery (MCA) within the CoW to replicate its anatomical complexity. This combined numerical–experimental methodology ensured reproducibility, physiological fidelity, and rigorous testing of device designs.

### Governing equations

The flow of blood and saline in the aspiration thrombectomy configurations was described by the standard incompressible continuity and momentum equations, written in unsteady form as follows^1^:

1 $$\frac{{\partial u_{i} }}{{\partial x_{i} }} = 0$$

2 $$\frac{{\partial \left( {\rho \overline{{ u_{i} }} } \right)}}{\partial t} + \frac{{\partial \left( {\rho \overline{{u_{i} }} \overline{{u_{j} }} } \right)}}{{\partial x_{j} }} = - \frac{{\partial \overline{p}}}{{\partial x_{i} }} + \frac{\partial }{{\partial x_{j} }}\left( {{\upmu }\frac{{\partial \overline{{u_{i} }} }}{{\partial x_{j} }} - \rho \overline{{u_{i}^{\prime } }} \overline{{u_{j}^{\prime } }} } \right)$$

where u is the velocity vector (m/s), p is the pressure (Pa), ρ is the blood density (1060 kg m^−3^), and ν is the kinematic viscosity (m^2^/s).

The non-Newtonian viscosity of blood was described using the Carreau–Yasuda model. This formulation was selected instead of the classical Carreau model because the additional Yasuda parameter provides greater flexibility in representing the transition between the low-shear Newtonian plateau and the high-shear shear-thinning regime. This is advantageous in the present aspiration-thrombectomy problem, where local shear rates vary substantially owing to pulsatility and venturi-induced acceleration. In addition, the Carreau–Yasuda model has been widely used in hemodynamic simulations of blood flow, including studies with experimental validation under physiologically relevant conditions^37,38^.

3 $$\mu \left( {x,t} \right) = \mu _{\infty } + \left( {\mu _{0} - \mu _{\infty } } \right)\left[ {1 + \left( {\lambda \dot{\gamma }} \right)^{a} } \right]^{{\left( {n - 1} \right)/a}}$$

where ${\mu}_{\infty }$= 0.0022 Pa s, ${\mu}_{0}$= 0.022 Pa s, $\lambda$= 0.11 s, a = 0.644, n = 0.392 according to Gijsen et al.^38^ and ${\dot{\gamma }}$ is the local instantaneous shear rate magnitude (s^−1^).

### Boundary conditions

**Fig. 1:**
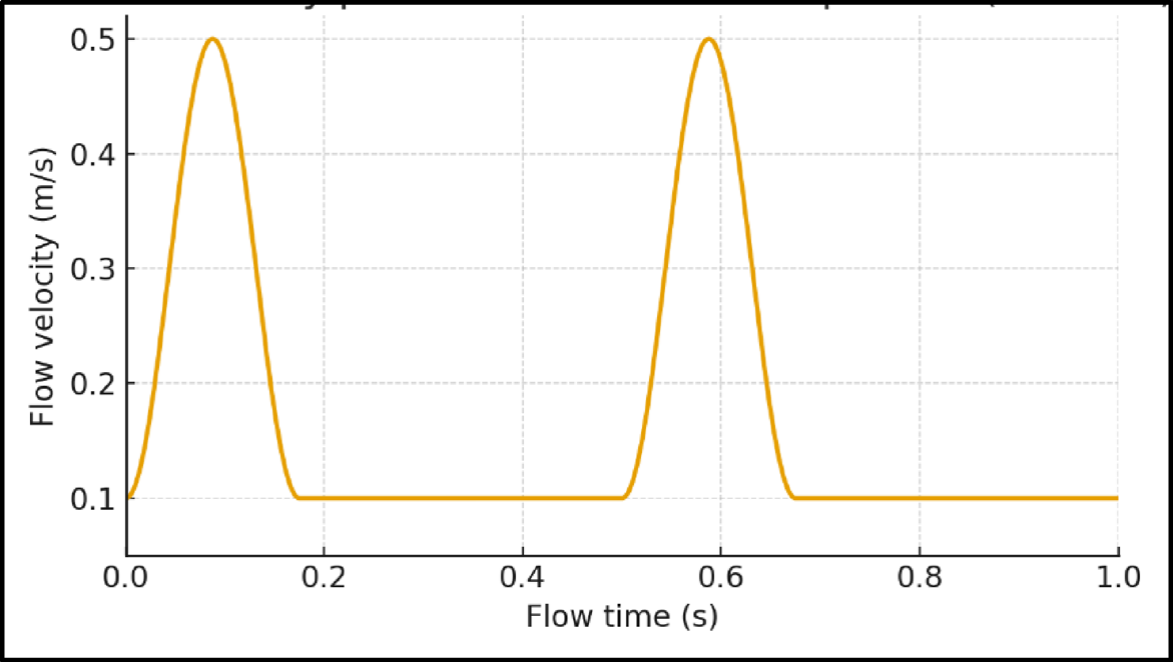
Inlet velocity profile.

At the inlet, a prescribed time-dependent axial velocity boundary condition was applied directly at the inlet plane of the computational domain. The temporal waveform was represented using a Fourier series with a fundamental frequency of 2 Hz (120 bpm). To preserve physiological waveform asymmetry, harmonics were retained up to the fifth order (≤ 10 Hz). The spatial velocity distribution at the inlet was assumed to be uniform across the inlet section of the simplified axisymmetric artery model; that is, the pulsatility was imposed through the time variation of the inlet velocity magnitude, rather than through an upstream inlet extension used to generate a developed profile. This choice was made to provide a controlled and identical inflow condition for all simulated device geometries in the present comparative study. The imposed waveform consisted of systolic and diastolic phases, with velocity varying between 0.1 m s^−1^ and 0.5 m s^−1^, as shown in Fig. 1. This choice is supported by reports that more than 90% of the pulsatile energy in the internal carotid and cerebral arteries is contained in harmonics below 6 Hz, with the dominant contributions occurring between 1 and 4 Hz^28,39,40^. This cyclic flow, with a period of 0.5 s, consists of systolic and diastolic phases. During systole, the velocity varies sinusoidally, peaking at 0.5 m/s and reaching a minimum of 0.1 m/s. This pulsatile profile is shown in Fig. 1. The mean Reynolds number, calculated using the generalized Metzner–Reed correlation, was approximately 345 for a vessel diameter of 4 mm, a value within the transitional regime and therefore supporting the adoption of a turbulence model^28^. A diameter of 4 mm was selected based on morphometric data of the Circle of Willis, where the proximal cerebral arteries typically exhibit diameters around this value^41^.

The outlet boundary was set to a constant suction pressure of –30 kPa, corresponding to the experimental vacuum system. Vessel walls were treated as no-slip boundaries, and near-wall resolution was handled using a boundary-layer inflation strategy adjacent to the solid walls. The thrombus was modeled as a 20 mm occlusion placed 3 mm downstream of the catheter tip. A 20 mm long thrombus with 4 mm diameter was positioned 3 mm downstream of the catheter tip, representing full occlusion of the vessel. The thrombus was represented as a highly viscous deformable occluding phase with density 1080 kg m^−3^ and dynamic viscosity 0.35 Pa s, according to Talayero et al.^42^. Within the present VOF framework, this treatment corresponds to a fluid-like clot surrogate rather than a solid-mechanics viscoelastic constitutive model.

### Computational domain and numerical details

**Fig. 2:**
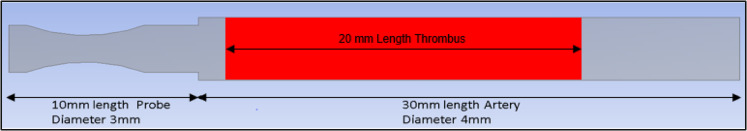
General computational geometry of the artery–device–thrombus domain used in the CFD simulations, showing the thrombus patch region. The detailed CAD geometries of the five device configurations are presented separately in Table 1.

The computational domain was constructed as a 2D axisymmetric geometry to reduce computational cost while preserving symmetry, since the aspiration device and vessel were coaxially aligned. Figure 2 illustrates the general computational arrangement of the artery–device–thrombus system used in the CFD simulations, whereas the detailed CAD geometries of the individual device configurations investigated in this study are provided in Table 1. The simulated artery was modeled as a 30 mm long straight segment with an internal diameter of 4 mm, corresponding to the silicone tubing used in the experiments. The aspiration device lumen was modeled with a diameter of 3 mm, attached coaxially to the artery. A 20 mm long thrombus with the same diameter as the artery (4 mm) was placed 3 mm downstream of the catheter tip, representing a 100% occlusion. An adapted interaction region was defined around the thrombus to capture the multiphase interface.

**Table 1:** The five designs of the aspiration based thrombectomy devices.

Name of design	Cross section of probe design	Description
30° Venturi curve		1cm length venturi probe with curved constriction at 30° to horizontal. It is curved to allow less friction and smoother flow
45° Venturi curve		1cm length venturi probe with curved constriction at 45° to horizontal. It is curved to allow less friction and smoother flow
60° Venturi curve		1cm length venturi probe with curved constriction at 60 degrees to horizontal Curved to allow less friction and smoother flow
7–11° Venturi straight		1cm length venturi probe with straight constriction. Entry at 11° and exit at 7° to horizontal. The angles have been investigated from an American Paper
Control device		The control device used to compare with other devices

An Eulerian-Eulerian approach within the Volume of Fluid (VOF) multiphase model was implemented in ANSYS Fluent 19.2 to simulate the multiphase interaction between blood and thrombus. The blood was defined as the continuous phase, with its non-Newtonian viscosity characterized by the Carreau–Yasuda law. The thrombus was modeled as the secondary phase and represented as a highly viscous deformable occluding phase with density 1080 kg m^−3^ and dynamic viscosity 0.35 Pa s. Thus, within the present VOF framework, the clot was treated as a fluid-like surrogate phase rather than as a solid body with a separately defined viscoelastic constitutive law. A geometric reconstruction scheme was utilized for the interface capturing to ensure sharp resolution of the blood-thrombus boundary. No interfacial surface tension term was included in the present VOF simulations; thus, no surface tension coefficient was prescribed. The interface dynamics were treated as being dominated by the imposed suction pressure, viscous effects, and geometry-induced acceleration within the simplified comparative modeling framework.

The pressure-based, transient solver was applied with the URANS framework and the standard k–ε turbulence closure. Pressure–velocity coupling was achieved using the SIMPLE algorithm. Spatial discretization was performed with a central-differencing scheme, and second-order implicit time stepping was used for temporal advancement. For the VOF model, explicit time integration was employed to track multiphase interactions.

Each simulation was run for eight cardiac cycles; the first three cycles were discarded to remove start-up transients, and the final five were used for analysis. Convergence was defined as residuals below 10^−5^ for velocity and continuity and below 10^−6^ for turbulent viscosity.

### Mesh independence

**Fig. 3:**
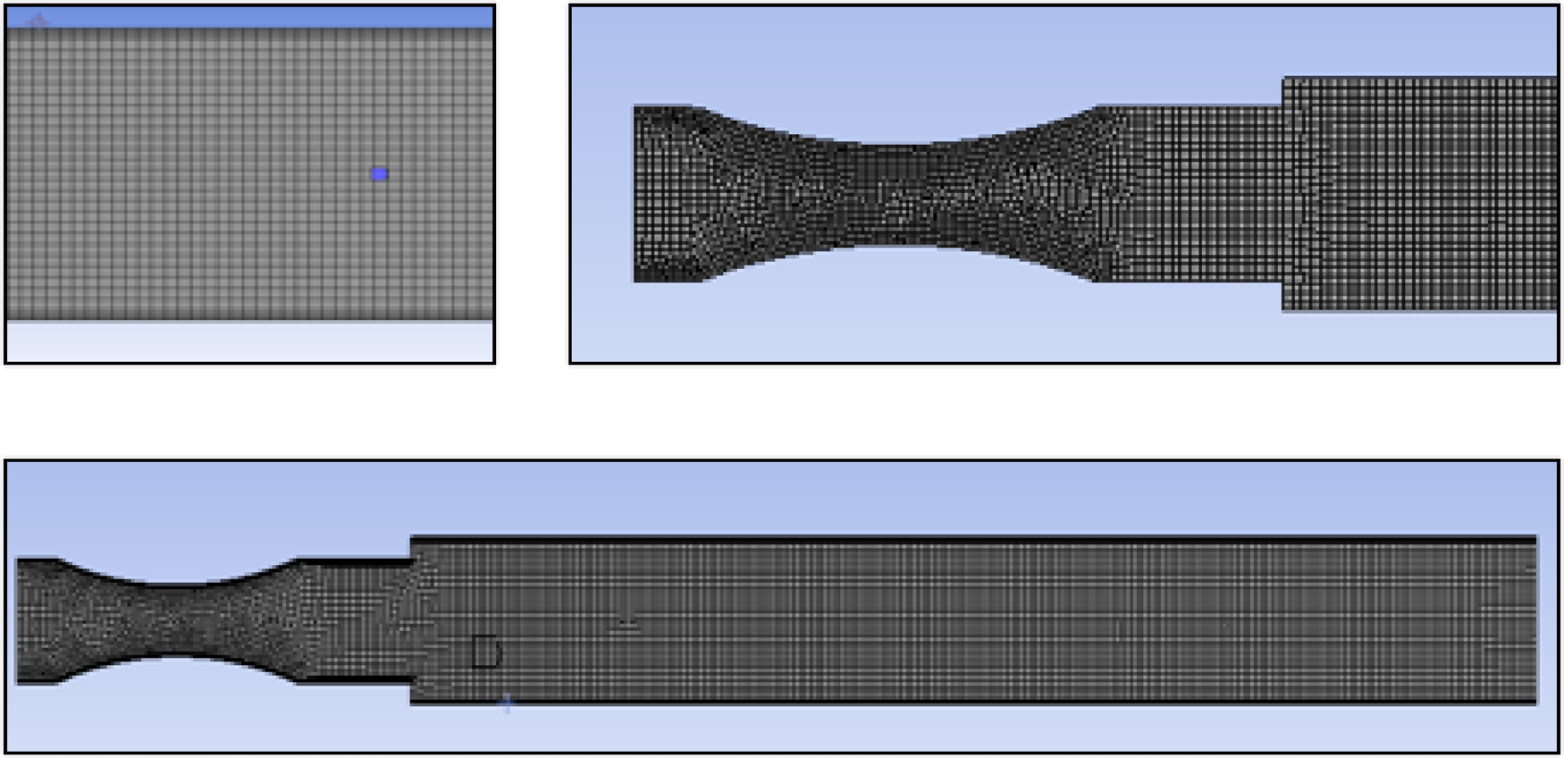
computational mesh of the artery–device–thrombus configuration used in the CFD simulations.

**Fig. 4:**
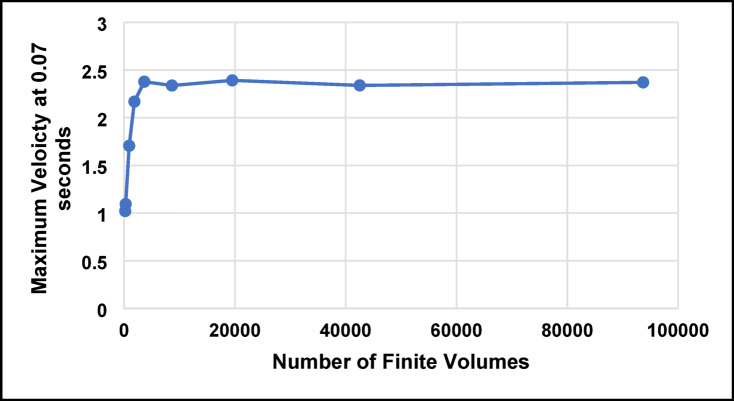
Mesh independence analysis for the 45° venturi geometry.

Grid independence was evaluated to ensure numerical accuracy while minimizing computational cost. The near-wall meshing strategy was kept consistent throughout the mesh-independence study. Specifically, the same wall-boundary treatment and inflation approach were retained for all mesh levels, and refinement was introduced through changes in the overall grid density rather than by altering the near-wall treatment itself. Due to the similarity of the venturi-style devices, the 45° geometry was selected as a representative case for the mesh refinement study. Simulations were performed with progressively refined grids, and the maximum velocity at the catheter tip was monitored at a representative timestep (0.07 s). Results showed negligible variation in velocity and shear stress beyond 4000 finite volumes, confirming mesh convergence. Accordingly, the final simulations were performed with 4000–5000 finite volumes, which provided a balance between accuracy and computational efficiency. The mesh convergence study yielded a maximum velocity of approximately 2.3 m/s, consistent with the expected suction-driven flow regime. Residuals were converged at 10^−5^ for velocity and continuity and 10⁻⁶ for turbulent viscosity, with a time step of 0.001 s ensuring numerical stability. Figure 3 shows computational mesh of the artery–device–thrombus configuration used in the CFD simulations. However, Fig. 4 represents the mesh independence analysis for the 45° venturi geometry.

### Device design and fabrication

Five designs of aspiration based thrombectomy device were investigated: three curved venturi designs with taper angles of 30°, 45°, and 60°, one straight taper geometry (7/11°), and a cylindrical control. Geometries were created in ANSYS DesignModeler 19.2.

**Fig. 5:**
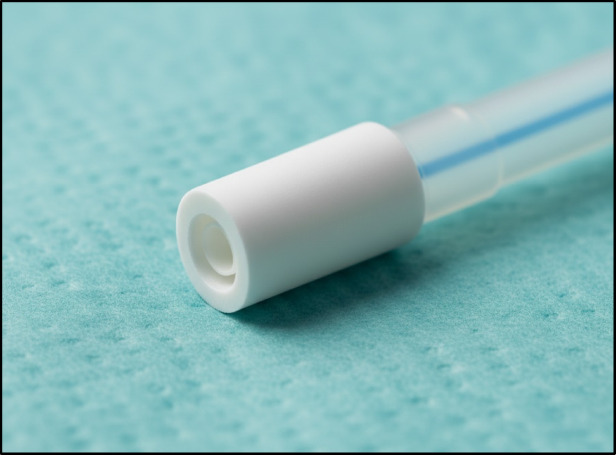
Printed device attached to rigid silicone catheter tubing.

Prototypes were manufactured using stereolithography (SLA) 3D printing with standard resin material to achieve high dimensional accuracy. The fabrication route was selected because the device geometry required a minimum wall thickness of 0.5 mm, which was compatible with the available SLA process. All prototypes were fabricated using the same manufacturing route and post-processing procedure, including washing, curing, and polishing of the tip region, to maintain consistency across the tested geometries. Each printed device was attached to rigid silicone catheter tubing, which provided sufficient support and prevented detachment during recanalization attempts, as shown in Fig. 5. Silicone was chosen over other common polymers such as nylon or polyurethane due to its inert properties and resistance to interaction with blood.

Table 1 shows the aspiration based thrombectomy device designs.

### Experimental setup

The experimental system was constructed to validate the aspiration performance of the venturi-inspired devices under physiologically relevant conditions. A soft silicone tube with a length of 150 mm and an inner diameter of 4 mm was used to represent a simplified cerebral artery. The material had a measured Young’s modulus of 1.68 MPa, consistent with vessel compliance reported in literature^43^.

N-saline solution was used as a substitute for blood. The solution was prepared by dissolving 9 g of sodium chloride in 1 L of water and mixing thoroughly. Prior to each trial, the interior wall of the silicone tube was lubricated with N-saline to facilitate clot insertion and reduce wall friction.

In addition to wall compliance, clot motion in the silicone phantom may also be influenced by surface friction and wettability at the clot–wall interface. In the present experiments, these effects were not independently characterized, but the same silicone material and the same N-saline pre-lubrication protocol were used throughout in order to maintain consistent interface conditions across all runs.

**Fig. 6:**
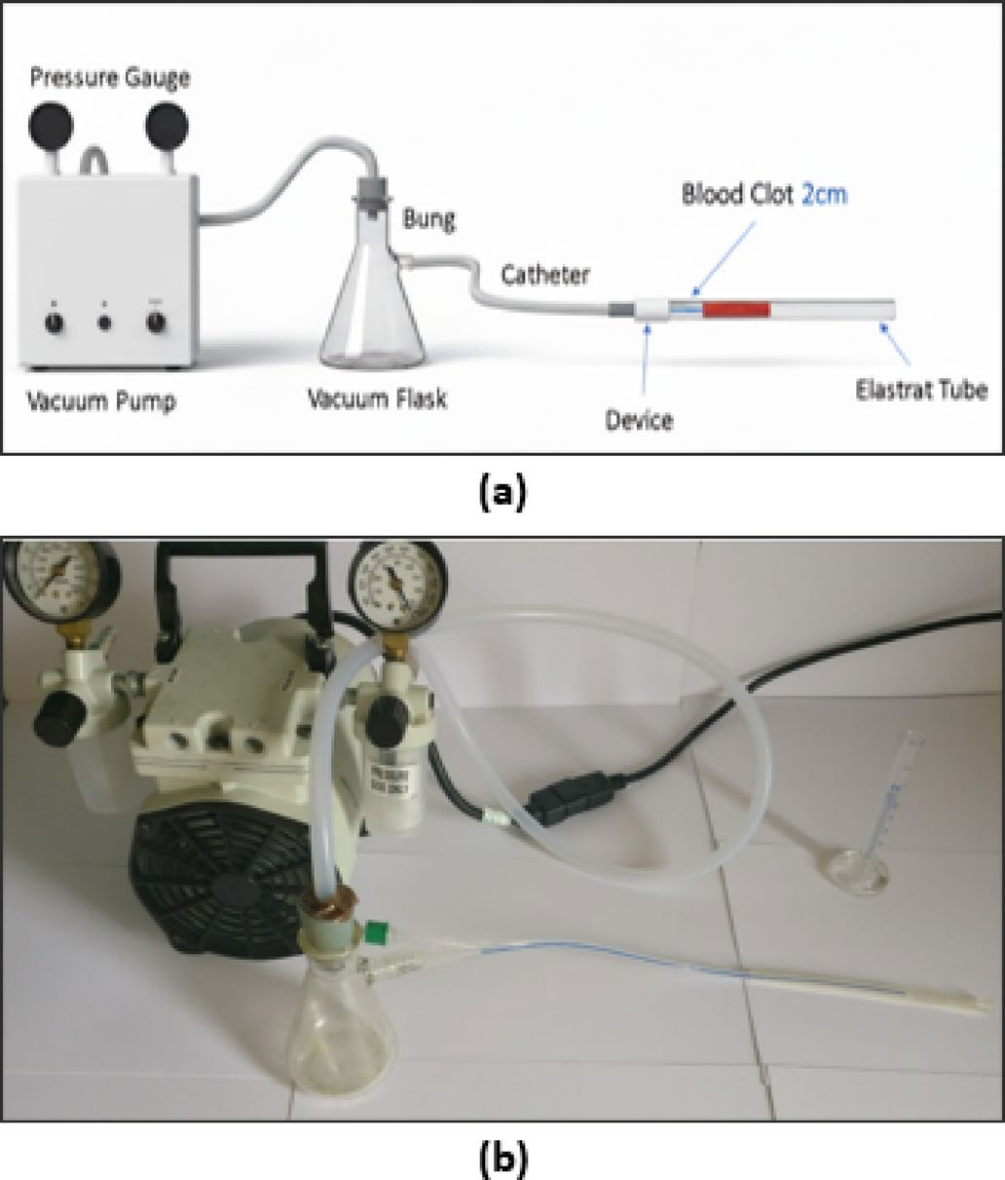
The experimental setup for in-vitro validation of venturi-inspired aspiration devices.

The aspiration system consisted of a vacuum pump connected via a PVC tube to a vacuum flask. A pressure gauge was integrated into the system and adjusted to maintain a constant negative pressure of − 30 kPa. The catheter was connected to the arm of the vacuum flask, with the probe inserted halfway into the silicone tubing to secure the assembly. Figure 6 illustrates both (a) the schematic diagram of the experimental setup for in-vitro validation of venturi-inspired aspiration devices and (b) a photograph of the real physical apparatus.

Cylindrical porcine clots, 20 mm lengths with 4 mm diameter, were prepared, induced with thrombin, incubated at 37 °C, and stored at 4 °C for 24 h before use. During each trial, a clot segment was inserted into the silicone phantom, with N-saline added to both ends of the tubing to ensure hydration and smooth insertion. The probe tip was then positioned 3 mm proximal to the clot before aspiration was initiated.

All in-vitro trials were conducted using the same experimental protocol, including identical nominal clot dimensions, the same silicone phantom geometry, a fixed probe-tip offset of 3 mm from the clot, N-saline lubrication of the tubing prior to insertion, and a constant applied suction pressure of − 30 kPa, in order to maintain consistency across replicates.

**Fig. 7:**
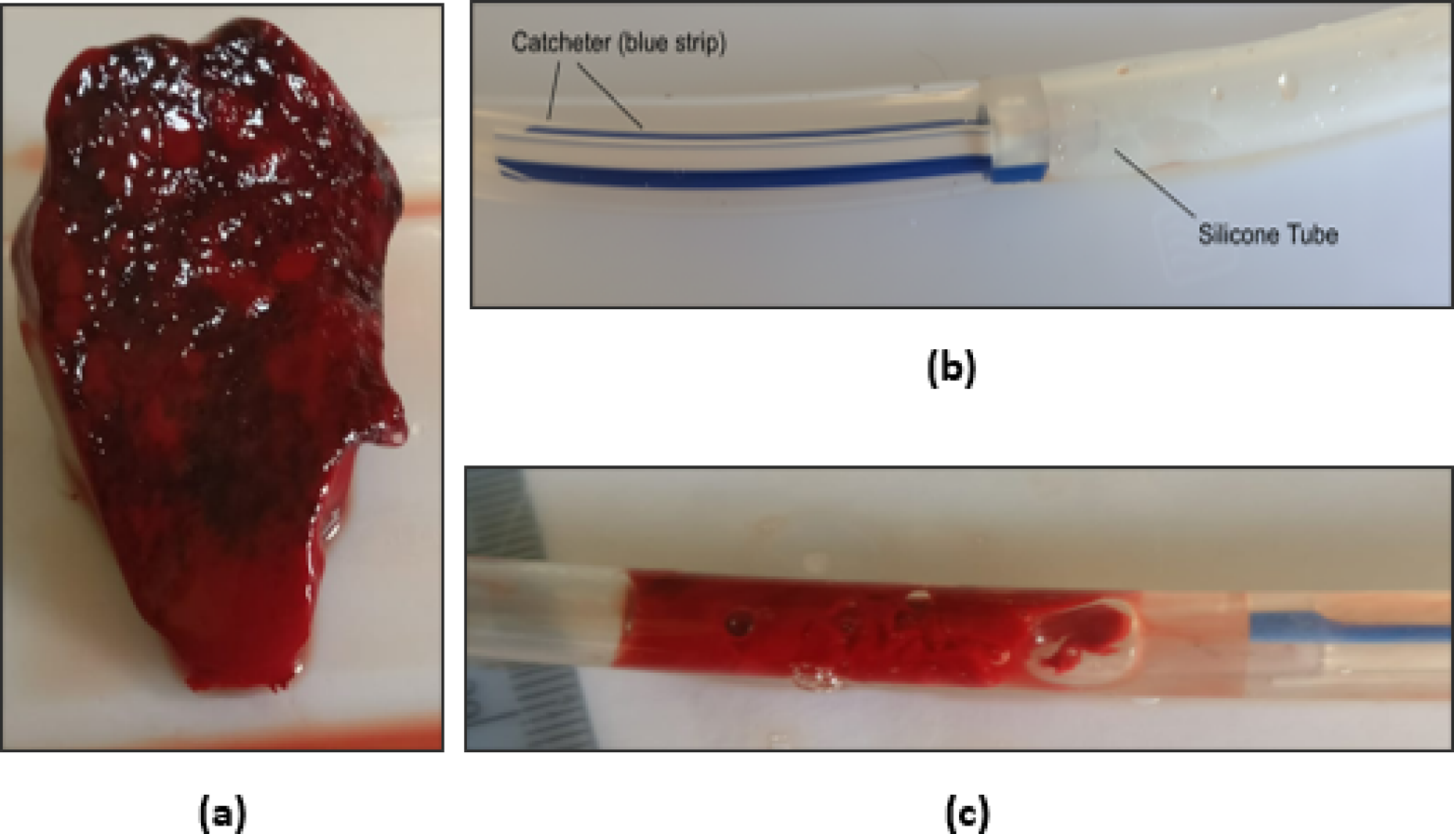
Porcine clot preparation and insertion into the experimental phantom. (**a**) Prepared cylindrical porcine clot. (**b**, **c**) Clot inserted into the soft silicone tube, representing the cerebral artery model.

Figure 7 shows the prepared porcine clot (a) and its insertion into the soft silicone tube (b, c), which was used to represent the simplified cerebral artery in the in-vitro setup.

Each device geometry was tested in ten replicates. The primary endpoint was the clot extraction time, defined as the duration required for ≥ 95% clot removal. Secondary endpoints included recanalization success rate, clot fragmentation, and residual clot length. In the present experiments, fragmentation was assessed qualitatively by visual inspection after each run. A fragment was defined as a visibly discrete residual clot piece remaining in the silicone phantom or at the device entrance region after aspiration, separate from the main clot body. Fragmentation was recorded as the number of such visible residual pieces per run; fragment mass was not measured.

The experimental work did not involve any live vertebrates or animal sacrifice. Porcine blood clots were prepared from commercially obtained porcine blood purchased from a licensed food supplier. All experiments were conducted in accordance with the relevant guidelines and regulations of Aston University (Birmingham, UK) and in line with the ARRIVE guidelines^44^ for studies involving animal-derived materials.

## Results

The performance of venturi-inspired thrombectomy devices was evaluated through in-vitro experiments and CFD simulations. In-vitro testing was conducted first to quantify clot extraction times, recanalization rates, and fragmentation across the five device geometries. These results were then complemented by CFD simulations, which provided detailed insight into suction velocities and pressure fields, and allowed direct comparison with experimental findings. Together, experimental and computational outcomes were used to assess device performance and to identify the optimal design.

### In-vitro evaluation of device performance

Prototype devices were fabricated by stereolithography and tested in vitro using porcine thrombi within silicone arterial phantoms, with ten independent replicates performed for each geometry (30°, 45°, 60° venturi, 7/11° taper, and cylindrical control).

**Fig. 8:**
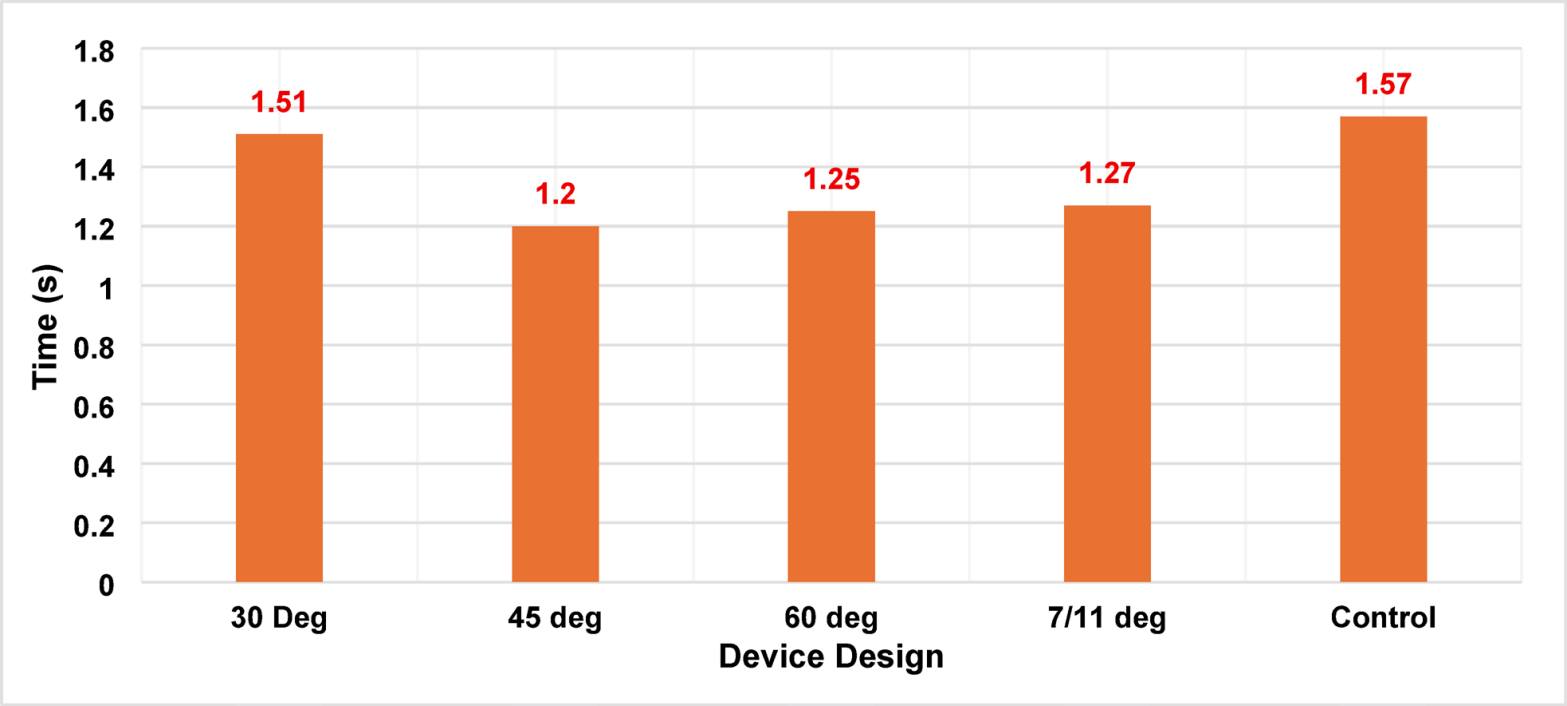
Mean extraction time for all venturi-inspired designs tested in-vitro. Error bars represent standard deviation across n = 10 replicates for each geometry.

In terms of extraction times, all venturi-inspired devices achieved faster clot removal than the cylindrical control, which required 1.57 s on average. The 30° venturi recorded the longest extraction time among the venturi designs at 1.51 s, while the 45° and 60° venturi achieved shorter times of 1.20 and 1.25 s, respectively. The 7/11° venturi (1.27 s) performed comparably to the 60° device, indicating that performance gains plateaued beyond 45°. These outcomes are shown in Fig. 8, which presents the mean extraction times for all devices.

To quantify experimental repeatability, the extraction-time data were further analyzed across the ten replicates for each geometry. The results were 1.51 ± 0.09 s for the 30° venturi, 1.20 ± 0.11 s for the 45° venturi, 1.25 ± 0.12 s for the 60° venturi, 1.27 ± 0.12 s for the 7/11° venturi, and 1.57 ± 0.19 s for the cylindrical control. The corresponding coefficients of variation (CVs) were 5.8%, 9.4%, 9.9%, 9.3%, and 11.9%, respectively. These values indicate moderate variability while preserving the same overall ranking across repeated trials, with the 45° venturi remaining the fastest design and the cylindrical control remaining the slowest. Table 2 shows the experimental repeatability metrics for clot extraction time (n = 10 per geometry).

**Table 2:** Experimental repeatability metrics for clot extraction time (n = 10 per geometry).

Device geometry	Mean extraction time (S)	Standard deviation (%)	Coefficient of variation (%)
30° Venturi	1.51	0.09	5.8
45° Venturi	1.20	0.11	9.4
60° Venturi	1.25	0.12	9.9
7/11° Venturi	1.27	0.12	9.3
Cylindrical control	1.57	0.19	11.9

**Fig. 9:**
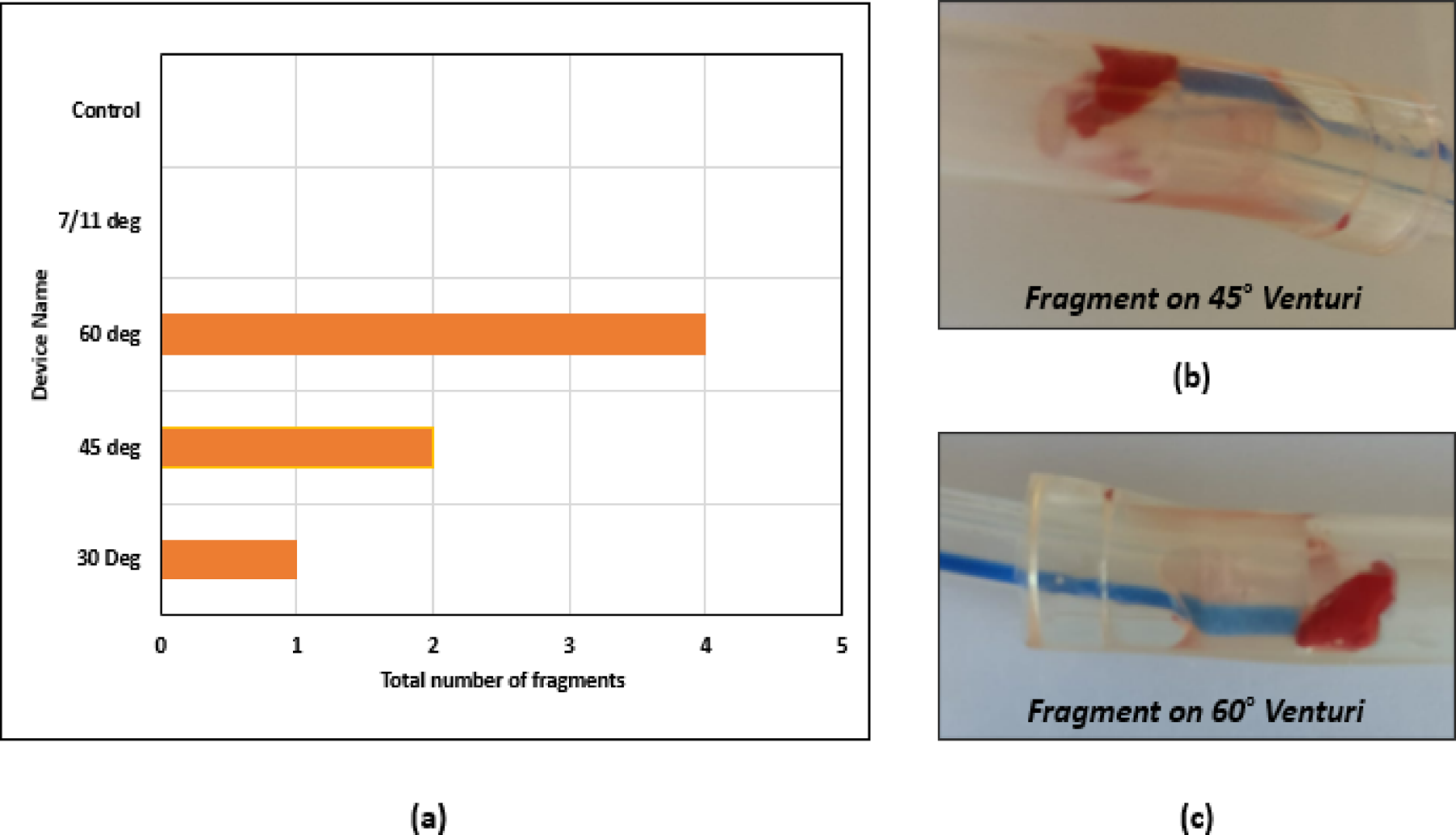
(**a**) Total number of visible residual clot fragments observed across n = 10 in-vitro runs for each venturi-inspired design. (**b**) Representative visible fragment observed after testing the 45° venturi device. (**c**) Representative visible fragment observed after testing the 60° venturi device.

Fragmentation analysis was treated as a qualitative count-based endpoint in the in-vitro experiments. The reported values represent the total number of visible residual clot fragments accumulated across the ten runs for each geometry. Under this definition, the 60° venturi generated four fragments, more than double the 30° design (one fragment), while the 45° venturi also produced visible fragmentation, as shown in the representative clot images. In contrast, the 7/11° taper and the cylindrical control produced no detectable fragmentation. In the observed fragmentation cases, the residual pieces were generally single visible fragments of approximately 1–3 mm characteristic size. These findings are illustrated in Fig. 9, which presents the fragment counts together with representative images from the 45° and 60° venturi experiments. Table 3 represents experimental fragmentation observations across n = 10 in-vitro runs per geometry.

**Table 3:** Experimental fragmentation observations across n = 10 in-vitro runs per geometry.

Device geometry	Runs with visible fragmentation (n/10)	Total visible fragments across 10 runs	Typical observed fragment size
30° Venturi	1	1	~ 1–3 mm
45° Venturi	2	2	~ 1–3 mm
60° Venturi	4	4	~ 1–3 mm
7/11° Venturi	0	0	None observed
Cylindrical control	0	0	None observed

### CFD simulations of aspiration devices

**Fig. 10:**
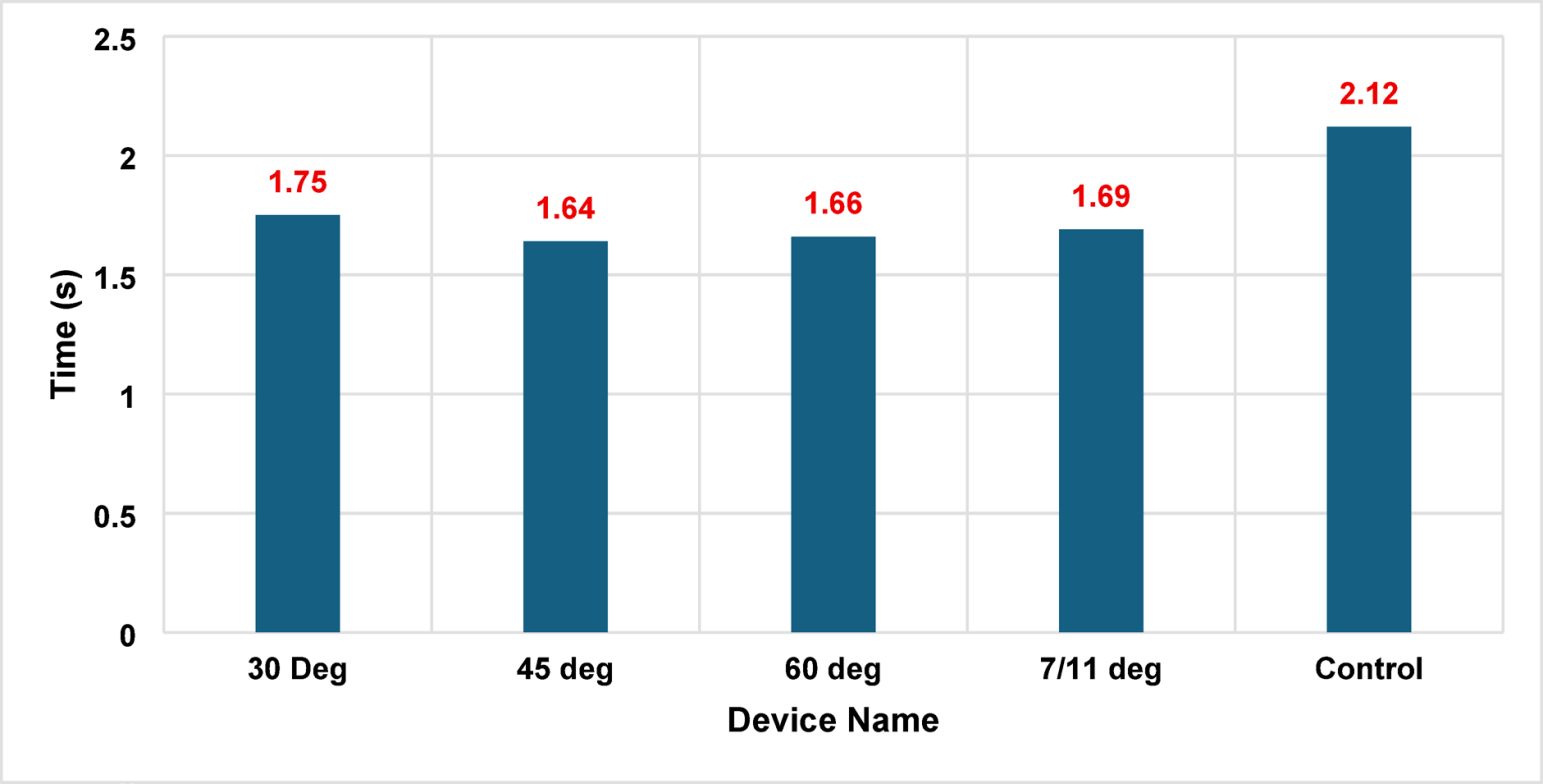
Extraction time for all aspiration device designs in CFD simulation.

CFD predicted extraction performance across the five venturi designs. The cylindrical control required 2.12 s for clot removal, markedly longer than all venturi designs. Among the venturi tapers, the 30° design was slowest at 1.75 s, the 45° design achieved the shortest time at 1.64 s, and the 60° and 7/11° designs recorded 1.66 s and 1.69 s, respectively. These outcomes are shown in Fig. 10, which summarizes CFD-predicted extraction times for all designs and highlights the plateau in performance beyond a 45° taper.

**Fig. 11:**
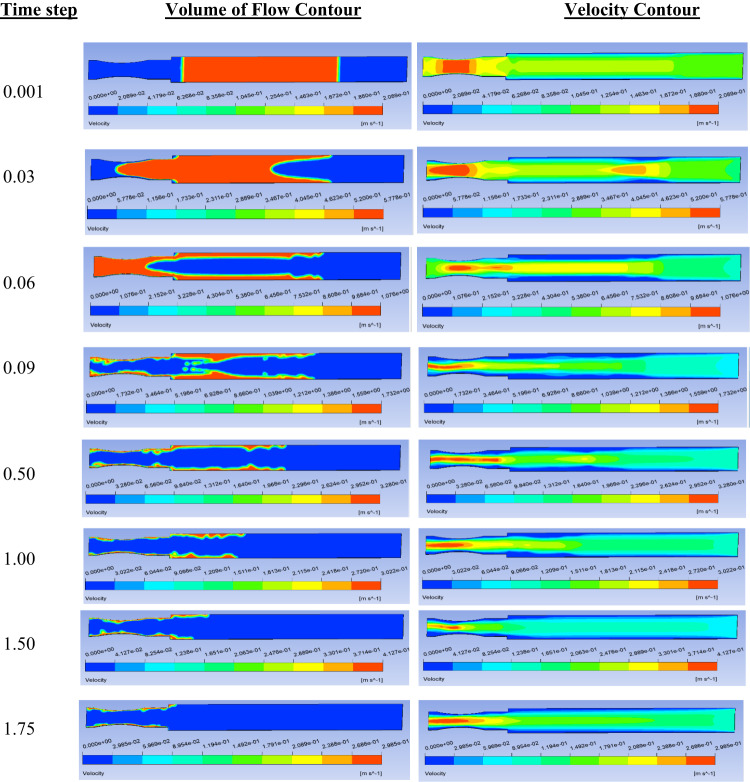
Volume of flow and velocity contours of 30° venturi-inspired device.

**Fig. 12:**
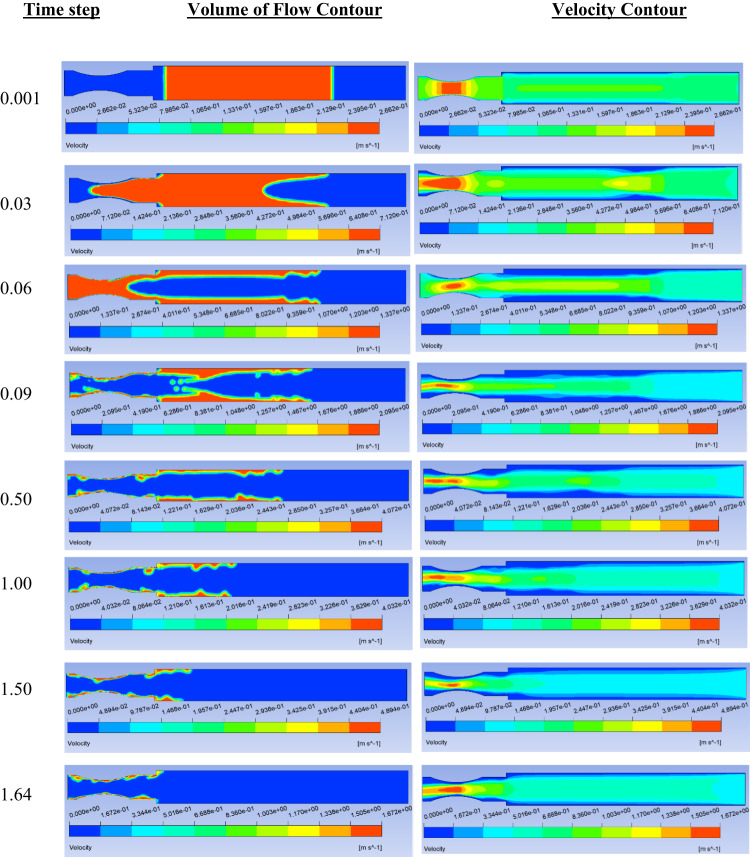
Volume of flow and velocity contours of 45° venturi-inspired device.

**Fig. 13:**
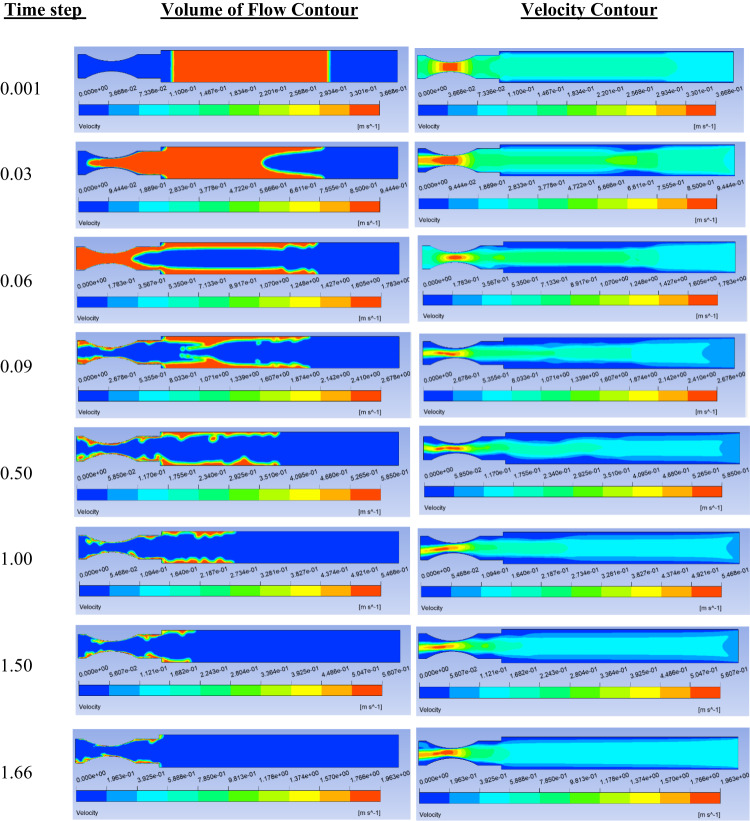
Volume of flow and velocity contours of 60° venturi-inspired device.

**Fig. 14:**
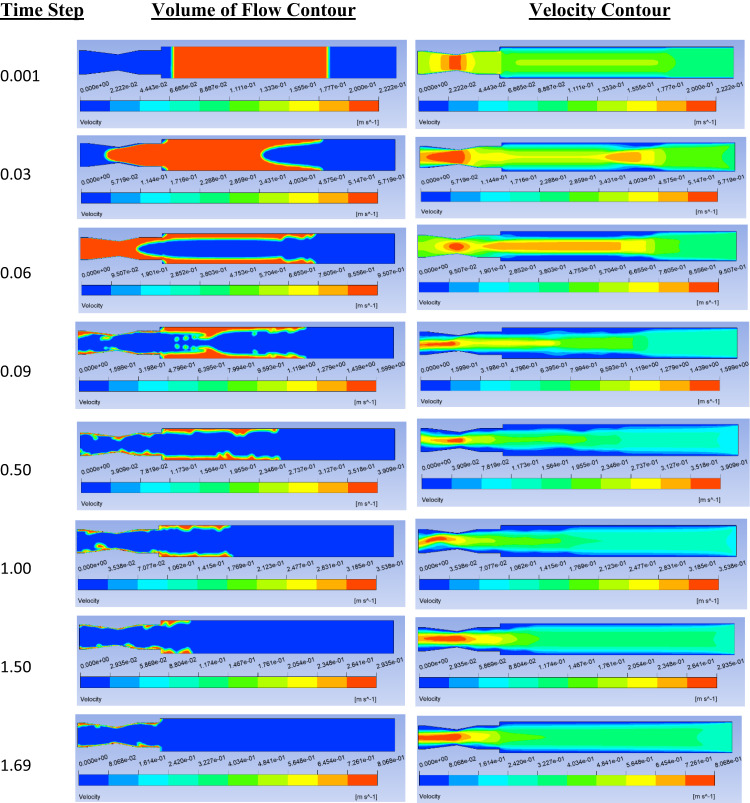
Volume of flow and velocity contours of 7/11° venturi-inspired device.

**Fig. 15:**
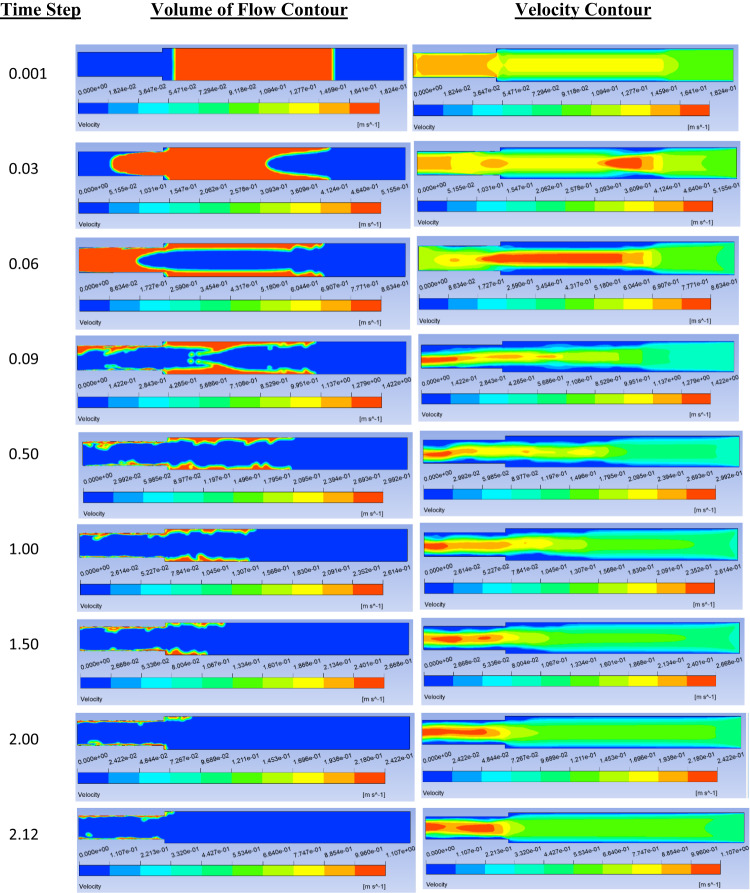
Volume of flow and velocity contours of cylindrical control venturi-inspired device.

Velocity and volume-flow contours provided qualitative insight into the flow-field differences underlying these trends. As illustrated in Figs. 11, 12, 13, 14 and 15, the venturi tapers produced a converging acceleration through the throat and a suction jet at the tip, whereas the cylindrical control lacked a convergent section and generated a more diffuse, lower-velocity field with reduced bulk flux toward the clot. The 60° venturi exhibited the most concentrated high-velocity core at the throat together with more pronounced localized shear-layer development near the tip, while the 45° venturi produced a similarly strong core with a more spatially confined disturbed-flow region. By contrast, the 30° taper exhibited weaker acceleration and a more diffuse jet directed toward the thrombus, while the 7/11° design generated the lowest jet intensity among the venturi tapers. Taken together, the extraction-time results and the velocity/volume-flow fields indicate that increasing taper angle intensifies local suction and jet velocity, whereas the performance gain becomes limited beyond 45°.Fragmentation was not quantified within the CFD framework; therefore, the simulated flow fields are interpreted only as qualitative hydrodynamic context for the experimental fragmentation observations.

**Fig. 16:**
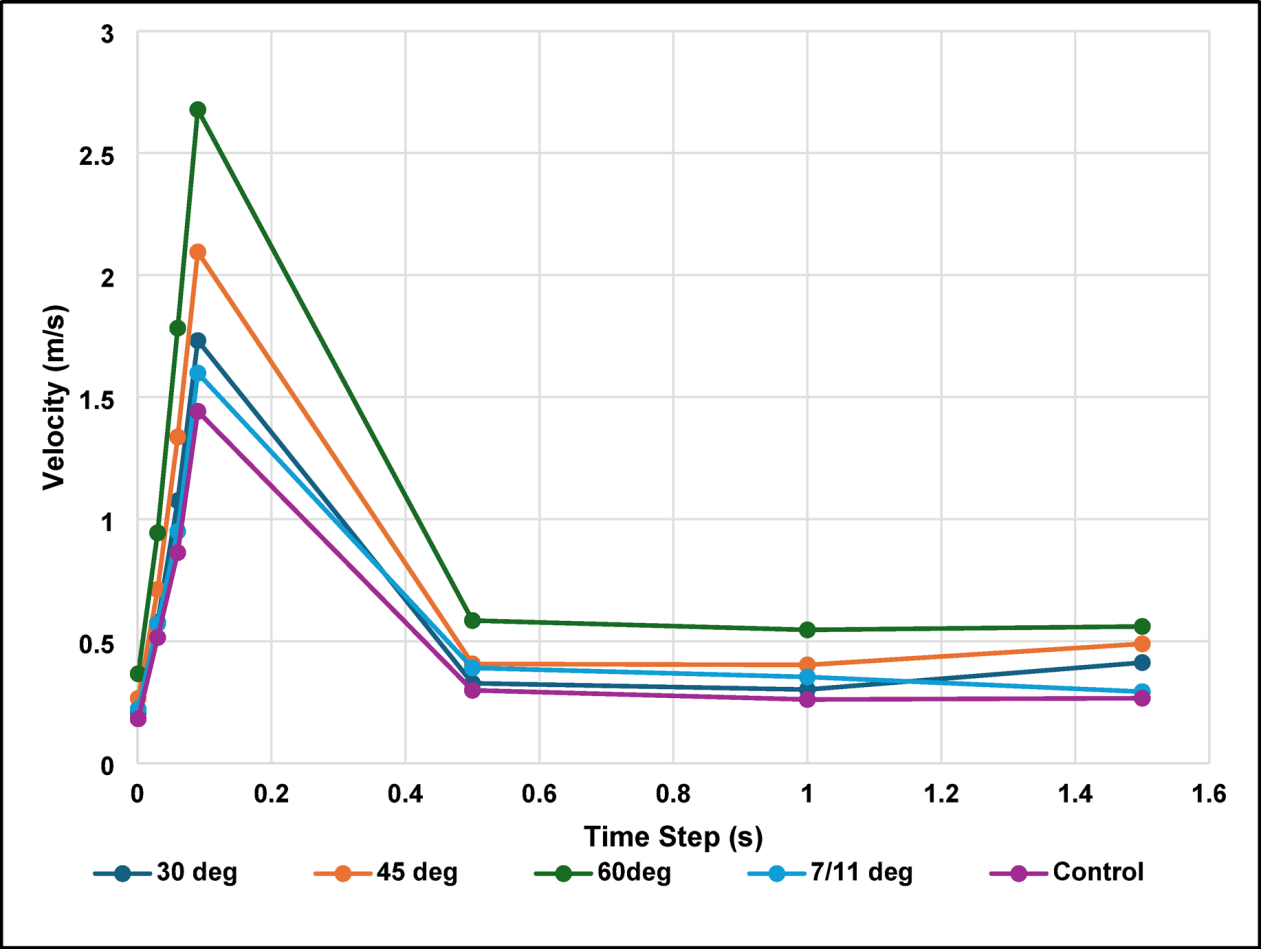
Peak-velocity comparisons across for all aspiration device designs in CFD simulation.

Peak-velocity comparisons across designs are summarized in Fig. 16. The 60° venturi produced the highest suction jet, exceeding the control by approximately 1.24 m/s at peak. Among the venturi tapers, the 7/11° design generated the lowest peak velocity (≈1.60 m/s), whereas the cylindrical control remained lowest overall when all designs are considered. Taken together, the extraction-time results and the velocity/volume-flow fields indicate that while increasing taper angle intensifies suction and local jet velocity, overall extraction efficiency plateaus beyond 45°, reflecting diminishing returns as increasingly concentrated disturbed-flow features develop near the catheter tip.

### Experimental validation of CFD

**Fig. 17:**
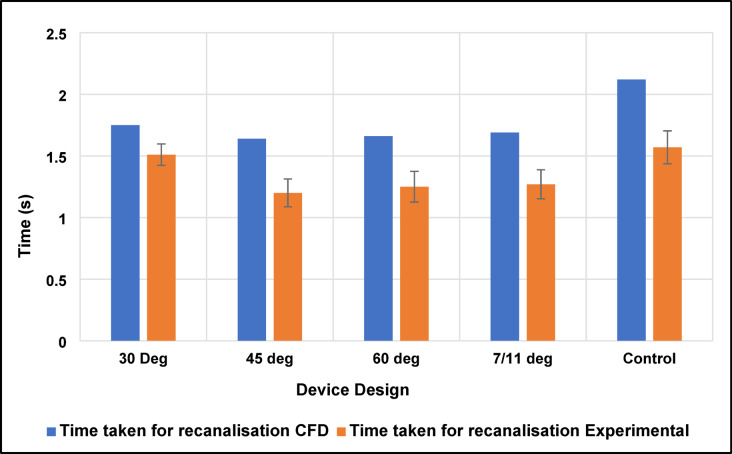
Extraction time comparison between CFD simulations and in-vitro measurements for all aspiration device designs.

Extraction times predicted by CFD were compared with the corresponding in-vitro measurements. As summarized in Fig. 17, both methods consistently identified the cylindrical control as the slowest design and the 45° venturi as the most efficient. CFD predicted removal times of 2.12 s for the control, 1.75 s for the 30° venturi, and 1.64 s for the 45° venturi, while experiments yielded shorter but similarly ranked values of 1.57 s, 1.51 s, and 1.20 s, respectively. The 60° and 7/11° venturi designs also showed close agreement, with CFD predicting 1.66 s and 1.69 s versus 1.25 s and 1.27 s in vitro.

Although CFD systematically overpredicted absolute extraction times relative to experiments, the rank ordering and performance plateau beyond 45° were reproduced consistently. This alignment confirms that the numerical model captured the essential flow–clot interactions governing device efficiency. The observed offsets are attributable to simplifications in the CFD setup, including rigid vessel walls, idealized clot rheology, and constant suction boundary conditions, in contrast to the compliance and pump dynamics present in the bench-top system. Despite these differences, the agreement between CFD and experiments supports the validity of the computational framework for comparative evaluation of venturi thrombectomy designs.

## Discussion

The present study combined CFD simulations and in-vitro experiments to evaluate venturi-inspired aspiration thrombectomy devices in a simplified cerebral artery model. The principal findings are that (i) both CFD and experiments consistently identified the 45° venturi as the most efficient design, (ii) extraction efficiency improved with taper angle up to 45° but plateaued thereafter, and (iii) higher venturi angles increased clot fragmentation, with the 60° design producing more than twice the fragments observed with the 30°. Importantly, the CFD predictions were validated by experimental outcomes: although absolute extraction times were systematically longer in simulations than in bench-top trials, the relative ranking of designs and the performance plateau beyond 45° were reproduced across both approaches.

### Principal findings and validation

The consistency between CFD and experiments strengthens the comparative evaluation of device designs, despite systematic offsets in extraction time. The CFD overpredicted removal times relative to experiments (e.g., 45° venturi: 1.64 s vs. 1.20 s), which can be attributed to several factors. First, the simulations assumed rigid vessel walls, whereas the silicone phantoms exhibited measurable compliance (E = 1.68 MPa), reducing resistance and facilitating faster clot removal. Besides compliance, the effective clot–phantom interaction may also depend on surface friction and wettability, which were not independently measured in the present study but were kept as consistent as practicable by using the same phantom material and lubrication procedure across all experiments. While these interfacial effects may influence the absolute clot-transport behavior, they are unlikely to alter the comparative ranking among the tested geometries under the controlled conditions used here. Second, the CFD boundary condition imposed a constant –30 kPa suction, while the experimental system used a vacuum pump with flask, introducing small fluctuations that enhanced aspiration efficiency. Third, the clot was modeled numerically as a uniform highly viscous occluding phase, whereas real porcine thrombi are heterogeneous and structurally more complex, often fragmenting earlier than the simplified numerical clot surrogate. Finally, the CFD solver employed a URANS k–ε framework, which does not resolve turbulence spectra directly. In the present study, the primary mechanisms supporting clot extraction are interpreted to be the venturi-induced pressure reduction, the resulting suction-jet acceleration, and the associated local shear acting at the clot–fluid interface. Prior work has reported that cerebral arterial flows may exhibit transitional and non-classical turbulent features, including non-Kolmogorov energy-transfer behavior under complex unsteady conditions^28^. In the present work, however, such phenomena are invoked only as a broader hemodynamic context for aspiration-induced flow unsteadiness, rather than as a directly demonstrated cause of clot detachment.

It should be emphasized that the CFD framework was validated against extraction-time trends and comparative flow behavior, whereas fragmentation was assessed exclusively in the in-vitro experiments and was not a directly simulated CFD output.

### Fluid-mechanical interpretation of venturi performance

The performance differences among venturi designs can be explained primarily by classical fluid-mechanical mechanisms, namely continuity-driven acceleration through the throat, Bernoulli-associated pressure reduction, and the resulting suction-induced loading at the clot interface. According to the continuity equation, the reduction in cross-sectional area through the venturi throat requires an increase in velocity to conserve mass flux. This acceleration is accompanied, by Bernoulli’s principle, with a reduction in static pressure at the throat, generating a suction effect that enhances clot aspiration.

In the present study, the 45° venturi achieved the best performance by combining strong throat acceleration with a comparatively stable suction jet directed toward the thrombus. The 60° design produced even higher peak velocities and lower throat pressures, but also exhibited a more concentrated disturbed-flow region and stronger local shear near the tip. These features provide only qualitative hydrodynamic context for the experimentally observed increase in fragmentation for the 60° geometry; however, the fragmentation trend itself was established experimentally, not predicted directly by the CFD framework. By contrast, the 7/11° taper produced smoother overall acceleration but a weaker suction jet, yielding slower extraction. The cylindrical control lacked a converging taper and therefore did not generate the same degree of acceleration or pressure reduction, consistent with its lowest velocities and longest extraction time.

These results confirm that the suction performance of aspiration catheters is governed by a balance between throat acceleration and downstream flow stability. Designs that maximize velocity while maintaining a stable suction jet, as demonstrated by the 45° venturi, achieve the most favorable experimental compromise between rapid clot extraction and limited visible fragmentation under the present test conditions.

### Relation to turbulence theory and hemodynamics

Beyond the classical Bernoulli and continuity mechanisms, the flow patterns observed in this study align with recent advances in cerebral hemodynamics that challenge the assumption of Kolmogorov turbulence. Saqr and Zidane^28^ demonstrated that arterial flows, particularly in intracranial arteries, can exhibit transitional TKE spectra with non-Kolmogorov features, including inverse energy cascades and anisotropy, which differ from classical turbulence assumptions. These regimes have been reported under conditions of pulsatility, complex geometry, and strong local flow disturbances.

In the present study, although URANS with k–ε closure cannot resolve turbulence spectra directly, the computed velocity and volume-flow fields revealed aspiration-induced flow disturbances and localized shear-layer development under pulsatile suction conditions. These features are interpreted only qualitatively in the present work. Prior studies have shown that blood flow in cerebral arteries may depart from classical laminar assumptions and may exhibit non-Kolmogorov characteristics under complex unsteady conditions^28^. Here, this literature is used as a physiologically relevant interpretive context, whereas the dominant determinants of clot extraction performance in the present results are taken to be mean suction velocity, Bernoulli-driven pressure reduction, and the resulting local hydrodynamic loading at the clot interface.

### Clinical and physiological implications

The middle cerebral artery (MCA) is among the most common sites of thrombo-occlusion in acute ischemic stroke, and its anatomy poses challenges for rapid and safe clot removal. The present findings suggest that venturi-inspired aspiration catheters with a 45° taper provide the most favorable balance between suction efficiency and clot integrity. The ability of this design to minimize extraction time while avoiding excessive fragmentation is particularly relevant clinically, since rapid recanalization correlates strongly with improved neurological outcomes, whereas clot fragmentation increases the risk of distal embolization and incomplete reperfusion.

The results further emphasize that device efficacy depends primarily on the hydrodynamic conditions generated at the catheter–clot interface, particularly suction intensity, throat acceleration, and local shear loading. The disturbed-flow features observed qualitatively in the 60° design, together with the experimental fragmentation trend, underscore the need for careful optimization of taper geometry. The finding that performance plateaued beyond 45° highlights the importance of balancing Bernoulli-driven acceleration with downstream flow stability, a consideration that may guide future catheter design.

By validating CFD predictions with in-vitro experiments, this study provides a framework for rational device optimization that bridges engineering performance metrics with clinically meaningful outcomes. Incorporating turbulence-aware modeling and clot–fluid interaction into the design process can improve the translation of computational insights into next-generation thrombectomy devices, ultimately contributing to safer and more effective stroke interventions.

## Conclusion and future directions

This study combined CFD and in-vitro experiments to evaluate venturi-inspired aspiration thrombectomy devices in a simplified cerebral artery model. Both approaches consistently identified the 45° venturi design as the most efficient geometry in terms of extraction performance, while the in-vitro experiments further showed that higher venturi angles were associated with increased visible fragmentation. Thus, under the present test conditions, the 45° design provided the most favorable experimental balance between rapid extraction and limited visible fragmentation. Although CFD systematically overpredicted extraction times relative to experiments, the agreement in rank ordering and performance trends validates the computational framework for comparative device assessment. The findings highlight the importance of taper geometry in shaping suction performance and demonstrate the value of a combined computational–experimental framework for thrombectomy device optimization.

Future work should extend the present framework in several directions. First, experiments and simulations should be performed in anatomically accurate Circle of Willis phantoms to account for branching, tortuosity, and collateral flow patterns. Second, compliant vessel models and more heterogeneous clot surrogates should be incorporated to better represent the mechanical environment encountered clinically. Third, higher-fidelity turbulence modeling such as large-eddy simulation (LES) may help resolve the non-Kolmogorov energy cascades reported in cerebral hemodynamics and clarify how aspiration-induced unsteadiness modifies the local flow environment near the clot interface. Finally, future prototypes should be fabricated using biocompatible flexible materials to enable clinically realistic navigation and aspiration testing.

By integrating computational and experimental insights, this work establishes a foundation for rational design of next-generation aspiration devices. Incorporating the outlined improvements will strengthen physiological fidelity and accelerate the translation of CFD-guided device optimization into improved outcomes for stroke patients.

## Data Availability

All data generated or analysed during this study are available from the corresponding author upon reasonable request.
